# Does midterm reinforcement improve compliance with farm-specific cow-comfort recommendations? Evidence from a nested randomized controlled trial in Kenyan smallholder dairy farms

**DOI:** 10.1016/j.vas.2026.100914

**Published:** 2026-10-24

**Authors:** Emmanuel Muunda, Elizaphan James O Rao, David C Hall, Caroline Ritter, Maria Camila Ceballos, John VanLeeuwen

**Affiliations:** a Faculty of Veterinary Medicine, University of Calgary, CWPH Building, 3280, Calgary, AB T2N 4Z6, Canada; b People, Policies, and Institutions Program, International Livestock Research Institute (ILRI), P.O. Box 30709, Nairobi, 00100, Kenya; c Department of Health Management, Atlantic Veterinary College, University of Prince Edward Island, Charlottetown, Canada

**Keywords:** Lameness, Implementation science, Animal welfare, Farmer behaviour, Veterinary extension

## Abstract

Farm-specific cow-comfort recommendations are often incompletely implemented. We evaluated whether midterm reinforcement increased implementation on Kenyan smallholder dairy farms and examined associations between achieved compliance and welfare and milk yield. Of 318 randomized farms, 212 received recommendations and 106 were controls. Recommendation recipients underwent a second randomization to reinforcement or no reinforcement (106 per group). Compliance was measured as weighted implementation fidelity (0–100%), combining recommendation effort and implementation status. Farm-level outcomes were compared by reinforcement assignment using available-case, covariate-adjusted models. Separate inverse-probability-weighted models estimated associations between achieved compliance (≥40% versus <40%) and outcomes. Among intervention farms with defined endline compliance scores, the covariate-adjusted difference by randomized reinforcement assignment was 12.0 percentage points (95% CI 2.7 to 21.3; p = 0.012), higher milk yield (1.33 kg/day; 95% CI 0.32 to 2.35; p = 0.011), and lower mean within-farm lameness prevalence (−19.75 percentage points; 95% CI −33.55 to −5.96; p = 0.005). In weighted analyses of achieved endline compliance, higher compliance was associated with lower endline lameness prevalence (-49.39 percentage points; 95% CI −60.64 to −38.15; p < 0.001), while the milk yield contrast (+1.00 kg/day) was not statistically significant (p = 0.121). Reinforcement assignment was associated with greater implementation, although incomplete follow-up limits inference. Higher achieved compliance was associated with lower lameness prevalence, but its nonrandomized, concurrent assessment with endline outcomes precludes a firm causal interpretation.

## Highlights

• Reinforcement assignment was associated with 12 percentage point higher endline compliance.
• Compliance was partial and varied widely across cow-comfort recommendations.
• Reinforcement assignment was associated with higher milk yield and lower lameness prevalence.
• Higher compliance was associated with lower endline lameness prevalence.
• Reinforcement may improve uptake of welfare-focused interventions in smallholder farms.

## Introduction

Cow comfort concerns the suitability of the housing environment for resting, moving, feeding and performing other normal behaviours without avoidable pain, injury or stress (Cook et al., 2005; von Keyserlingk et al., 2009). Cow comfort is strongly dependent on housing and stall design, stall dimensions and flooring characteristics, availability and type of bedding and day-to-day stall management, among other factors. Recent research in dairy production has identified cow comfort as one potential leverage point for improving welfare and performance of dairy cattle on smallholder farms (Kariuki et al., 2023).

Across livestock systems, farmers are regularly given evidence-based recommendations on targeted, farm-specific adjustments that would improve cow comfort on their farms and consequently realize benefits that accrue from improved animal welfare. These benefits largely depend on the extent to which farmers comply with and sustain the recommended adjustments. While such recommendations can produce meaningful improvements in knowledge and intentions (Ledo et al., 2021), they frequently fail to deliver their full benefits because they do not necessarily translate into sustained implementation (Kathambi et al., 2019). Feasibility concerns, doubt about the effectiveness of the recommendations, competing priorities and the practical challenge of sustaining routine management adjustments in the long run have been reported as major constraints to comprehensive uptake of the recommendations (Kathambi et al., 2019; Ritter et al., 2017).

The gap between farmers’ knowledge and intention and their actual actions has been widely recognized across agricultural and veterinary extension in diverse contexts. In high income settings, for instance, a qualitative study of calf-health advice on nine German dairy farms found that 15 of 36 (42%) proposed measures were implemented over one year (Douay–Ryckelynck et al., 2025), with inadequate team capacity, perceived effectiveness and limitations of existing facilities being reported as key barriers. Recommendations that required recurring implementations were less likely to be implemented. Similarly, another study reported that some farmers did not feel ready to implement provided recommendations even with routine veterinary advisory visits (Ritter et al., 2019).

The challenge of incomplete implementation is likely amplified in low- and middle-income countries, where resource constraints, labour limitations, and weak market incentives constrain the ability and motivation of farmers to adopt cow comfort recommendations fully (Muunda et al., 2026). In sub-Saharan Africa and Southern Asia, dairy farming is predominantly smallholder and a common source of household livelihoods, but often, smallholder dairy farmers operate under narrow margins with high exposure to risk. In Kenya specifically, the dairy sector contributes 14% of agricultural GDP, and smallholder farms account for 70% of the country's milk production (World Bank, 2021). At the same time, 80% of all the production is estimated to be marketed through informal milk markets, where price signals and compliance incentives (improved cow comfort standards in this case) are often limited. Such realities make animal welfare and productivity recommendations attractive in principle, but difficult to fully and consistently implement in practice, even when low or no cost is involved.

Previous randomized controlled trials (RCTs) in Kenya delivering farm-specific cow comfort recommendations have consistently observed variable and partial compliance. Kathambi et al. (2019) reported that 46 of 62 intervention farmers made at least one change and implemented only 63% of issued recommendations. Kariuki et al. (2023) reported an overall compliance rate of 49%. Ng’ang’a et al. (2025) observed that a mean compliance of 2.1 on the cow comfort recommendations and 2.8 on the mastitis control recommendations, producing a 63.2% overall compliance, further implying both low average compliance and substantial heterogeneity between farms.

Incomplete implementation may limit the benefits achievable from farm-specific recommendations. First, it may reduce the potential impact of the intervention received, thereby reducing the effect of the expected improvements of cow comfort, cleanliness and health outcomes, even when the provided recommendations are technically correct. Secondly, the heterogeneous compliance potentially introduces equity questions: if some farmers adopt most recommendations and others adopt a few or none, the benefits of advisory/extension interventions may concentrate among farmers with greater resources, higher motivation and fewer constraints – likely widening performance gaps. For instance, Kathambi et al. (2019) reported differences in implementation according to recommendation type and the person receiving the advice. These findings support evaluating implementation directly alongside animal outcomes.

A reinforcement visit provides an opportunity to review implementation, clarify recommendations and discuss difficulties encountered by farmers. These activities are consistent with the Knowledge-to-Action framework, which includes monitoring knowledge use and addressing barriers to implementation (Graham et al., 2006). Reinforcement has been found to provide policy-relevant approaches to increasing and sustaining adoption (Blekking et al., 2026). Whether reinforcement improves implementation of cow-comfort recommendations in this setting requires direct evaluation.

Existing literature from agricultural extension research has reported that repeated engagement with farmers can improve adoption. Evidence from an extension network study in Mozambique showed that providing additional training as a way of augmenting standard information diffusion could increase adoption among targeted farmers (Kondylis et al., 2017). In another study by Blekking et al. (2026), they observed that well-timed reminders (including mobile-based text message reinforcement) can help maintain farmer engagement and increase adoption of recommended practices beyond the initial training. In livestock extension, feedback tools, such as benchmarking, have also been found to strengthen cooperation between veterinarians and farmers, and strengthen the latter’s perception of veterinary advisory capacity, supporting the idea that reinforcement can shift advisory interventions from one-off recommendation to sustained change processes (Sumner et al., 2020).

The implementation research gap addressed here is whether a structured midterm reinforcement visit increases compliance beyond that achieved after the initial provision of farm-specific recommendations. The present study evaluated a randomized trial among smallholder dairy farms in Uasin Gishu County, Kenya and included a second randomization to reinforcement within the group receiving recommendations. The study’s objectives were twofold: (i) to estimate the effect of assignment to midterm reinforcement on endline compliance; and (ii) to assess the association between achieved compliance and endline cow-welfare outcomes and milk yield.

## Materials and methods

### Study area

This study took place in Uasin Gishu County in Kenya, East Africa. Uasin Gishu is located along the equator, to the North of Kenya's Rift Valley region, approximately 320 km N.W. of the country’s capital, Nairobi. The county has a population of 1.16 M people and covers a land area of approximately 3300 km^2^. It is a high-altitude area with moderate temperatures and bimodal annual rain patterns, and one of the country’s most productive agricultural regions for both livestock and crop production.

### Study participants and sampling

The study population consisted of smallholder dairy farms (farmers keeping 5 or fewer cows) practicing zero-grazing or semi-zero-grazing systems, as these systems are directly affected by stall-based cow comfort recommendations. Farms were considered eligible if the household owned at least one lactating cow that had not been bred for more than three months prior and expected to be dried off within six months after enrolment. Exclusion criteria included farms without stalls for cows and farms with more than five cows (no longer smallholder).

Following (Dohoo et al., 2014), the required sample size calculation was based on detecting a minimum effect size of 15% improvement in compliance with cow comfort recommendations, assuming 80% statistical power, a two-sided 5% significance level, and an allocation ratio of 2:1 (treatment: control). The resulting total analysable sample of 270 farms was calculated based on plausible variabilities of compliance scores observed in previous studies in similar contexts (Kariuki et al., 2023; Kathambi et al., 2019). Based on the above factors, and accounting for a potential attrition risk of 15%, 318 dairy farms were recruited.

### Study randomization and design

This study adopted a two-stage randomized controlled trial design. Eligible farmers were first randomized to intervention (n = 212) and control (n = 106) groups. At the mid-term point of the RCT, a computer-generated random allocation sequence in STATA version 18, was used to assign intervention farms to either (i) treatment with a midterm reinforcement visit (n = 106), or (ii) treatment without reinforcement (single provision of recommendations) (n = 106). Because compliance with the farm-specific recommendations was only meaningful among farms that received recommendations, the primary compliance analysis compared reinforcement assignment groups among intervention farms with observed and defined endline compliance scores. Farms were analysed according to their assigned group, irrespective of whether they received reinforcement.

During the baseline visit, all farmers consenting to participation underwent a comprehensive assessment covering husbandry practices and related costs, stall dimensions, select-cow welfare indicators, and production parameters. Cow-level assessments included California Mastitis Test (CMT) results, lameness scoring, lesion scoring, body condition scoring, and cow hygiene scoring, using standardized field protocols previously applied or adapted for dairy welfare and comfort assessment (Cook et al., 2005; Kariuki et al., 2023; Reneau et al., 2005; Sprecher et al., 1997). All farmers randomized to the treatment arm received farm-specific cow comfort recommendations tailored to their baseline assessment, focusing on low-cost “light-touch” interventions, as described elsewhere (Kathambi et al., 2019; Kariuki et al., 2023).

The midterm visits were implemented approximately four months after baseline among farms assigned to the reinforcement arm. During these visits, the study team reassessed cow comfort status using the same farm-specific recommendation checklist provided during the baseline visit. For each recommendation, the assessor reviewed whether the action had not been implemented, had been implemented partially or fully. Farmers were then given item-specific feedback, including acknowledgement of completed actions, clarification of incomplete or incorrectly implemented tasks, and a discussion of remaining areas where comfort adjustments were required. Reinforcement therefore entailed structured feedback, reminders of the initial recommendations and discussion of implementation difficulties. It did not introduce a new recommendation package. Farmers and the staff delivering recommendations and reinforcement were aware of the intervention activities.

Endline assessments were scheduled approximately six months after baseline and were completed at similar follow-up intervals across trial arms. These assessments prioritized control group farms with a median time of 178 days from baseline to endline (IQR 176 – 181), 181 days for intervention farms not assigned to reinforcement (IQR 177 – 185), and 180 days for intervention farms assigned to reinforcement (IQR 176 – 185). For farms receiving reinforcement, the median time from baseline to reinforcement was 120 days (IQR 116 – 124), and 60 days (IQR 56 – 64) from reinforcement to endline. Farmers in the control arm received farm-specific cow comfort recommendations during endline visits as a delayed intervention, ensuring equitable access to the intervention package after their role as controls in the trial was complete. Fig. 1 presents the schematic flow of how the study was implemented.

**Fig. 1:**
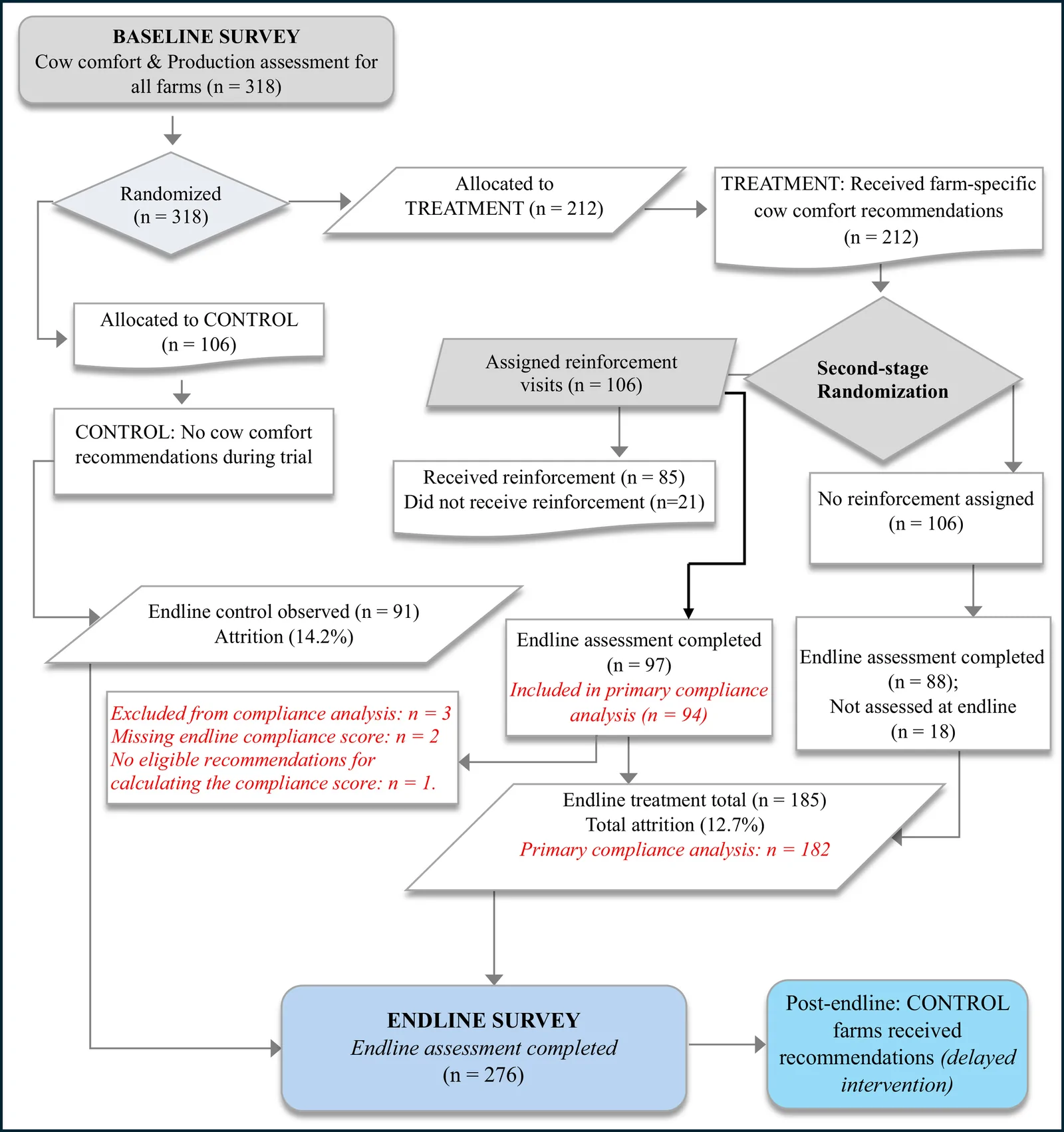
Farm allocation, reinforcement receipt, endline follow-up and inclusion in pimary analysis.

### Data collection

All farms underwent comprehensive cow comfort and production assessment to: (a) characterize existing cow comfort conditions and management practices, and (b) generate farm-specific recommendations for treatment farms. Data were collected through structured questionnaires administered to farmers using Open Data Kit, a Computer-Assisted Personal Interviewing software on tablet devices. The interviews were conducted in English, Swahili, or Kalenjin, depending on the respondent’s language preference. Before field implementation, research assistants were trained on the structured cow-comfort assessment protocol, including stall measurements, stall-condition ratings, animal-based welfare scoring, hygiene scoring, lameness scoring, body condition scoring, lesion scoring, and California Mastitis Test procedures. Training included review of standardized scoring criteria and pictorial scoring guides for body condition, lameness, udder hygiene, and leg hygiene, followed by pilot farm assessments to harmonize interpretation of scoring categories and field procedures. The same predefined scoring categories were used across all visits to promote consistency across assessors and survey rounds. The same observers were used throughout the study. Formal inter-observer agreement statistics were not calculated.

### Cow comfort assessment

The baseline, midline and endline questionnaires included modules relevant to the objectives of this study, including stall design, stall dimensions and management. These questionnaires also included management data capturing farmer-reported practices relevant to maintaining a clean, dry, and soft lying area, including the frequency of manure removal from the lying area and alleyway, whether bedding was used (and the bedding type), and how often wet bedding was removed and new bedding was added (Cook et al., 2005). In parallel, the study team conducted standardized observations of the lying surface and surrounding stall environment, recording the stall-floor type (e.g., dirt vs. concrete), whether the floor was lumpy/uneven, whether fluids (e.g. rainfall) could drain under the udder area, stall wetness rating, stall softness rating, the presence of sharp objects that could injure cows, roof adequacy (coverage and holes), and the alleyway condition, as described elsewhere (Kathambi et al., 2019; Kariuki et al., 2023).

Cow-level welfare and cleanliness indicators were assessed for each eligible milking cow, with emphasis on measures that are sensitive to stall design and stall-surface management (Reneau et al., 2005). Stall-use and comfort-related indicators (e.g., whether cows bumped into wood railings when rising, lie down outside the stall, or showed “odd stall use” such as perching or standing idle), and scored injuries/lesions at the neck, hock, and carpus were recorded using categorical severity classes (Cook et al., 2005). Hygiene was assessed for udder hygiene and leg hygiene using ordinal scales. Lameness was scored through visual assessment of gait (Sprecher et al., 1997), alongside basic animal descriptors (breed, weight) that informed cow-weight–adjusted interpretation of stall dimensions. California Mastitis Test reactions were scored at the quarter level (Reneau et al., 2005).

Finally, stall infrastructure was evaluated through direct measurements and structured adequacy ratings aligned with the approach described by Kariuki et al. (2023). Stall dimensions and design elements were assessed for each stall, including total width, total length, body length, brisket-board status, neck-rail status, forward/side lunge space, and leg space, and these assessments were categorized using a three-level judgement (good/adequate, marginal, not good/inadequate) (Cook et al., 2005). The study tool operationalized these three categories using cow-weight–referenced cut-offs for key dimensions (e.g., “good” when within ∼10% of the expected value; “marginal” when ∼10–20% away; and “not good” when >20% away). For analysis, marginal and not-good ratings were combined as a marginal-or-worse deficit category, identifying stalls that did not meet the study’s “good/adequate” criterion. Throughout the manuscript, “inadequate” refers to this combined category. The lying surface itself was further assessed using practical field tests analogous to those described by Kariuki et al. (2023): a knee impact assessment to reflect stall-base hardness and a knee wetness assessment (based on the level of pain when dropping to one’s knees from a crouch, and on whether knees were dry/slightly wet/wet after kneeling for ∼20 s in the udder area, respectively), alongside structured ratings of stall hygiene.

### Analytical framework

The analyses addressed the two study objectives by examining compliance first as an outcome of reinforcement assignment and then as an exposure associated with animal welfare and productivity. For the first objective, endline compliance scores were compared between intervention farms randomly assigned to receive midterm reinforcement and those assigned to receive no reinforcement. Additional analyses compared endline milk yield and welfare outcomes between these assignment groups. Farms were analysed according to their randomized assignment, with each analysis restricted to farms with the required outcome and covariate data.

For the second objective, endline milk yield and welfare outcomes were compared between intervention farms with compliance scores ≥40% and those with scores <40%. These groups were defined by achieved compliance rather than randomized allocation. Inverse-probability weighting was therefore used to account for measured baseline differences between the compliance groups. These analyses estimated associations between achieved compliance and outcomes; their interpretation remains subject to residual confounding. Table 1 summarizes the construction of the compliance measure and the analyses addressing these objectives.

**Table 1:** Overview of compliance scoring and analyses addressing the study objectives.

Component	Population and comparison	Outcome or purpose	Analysis
Compliance scoring	Intervention farms with eligible recommendations	Implementation relative to each farm’s recommendation package	Effort-weighted score normalized to 0–100%
Reinforcement and compliance	Reinforcement versus no reinforcement assignment; farms with defined endline scores	Endline compliance	Unadjusted and adjusted linear regression; fully interacted sensitivity model
Reinforcement and animal outcomes	Intervention farms with observed outcome data, analysed by reinforcement assignment	Milk yield and welfare outcomes	Baseline-adjusted linear models; fractional-logit sensitivity models for prevalence outcomes
Achieved compliance and outcomes	Intervention farms with defined compliance scores; compliance ≥40% versus <40%	Endline milk yield and welfare outcomes	Inverse-probability-weighted association models
Paired implementation	Reinforcement recipients observed at midline and endline; item-specific samples	Any implementation of each recommendation	Paired proportions and McNemar tests
Diagnostics and sensitivity analyses	The relevant analytical samples	Weight distributions, measured-covariate balance and dependence on model specification	Reported diagnostics and sensitivity comparisons

#### Analysis populations and descriptive summaries

The baseline farmer, farm-level characteristics and cow comfort status were summarized by randomized arm. Continuous variables were described using means and standard deviations when approximately symmetric and medians with interquartile ranges when skewed. Categorical variables were summarized as counts and percentages. Baseline characteristics were described by randomized group without significance testing.

Cow-level measures were aggregated to the farm level before arm-level comparison because the farm was the unit of randomization. Continuous cow-level outcomes, such as milk yield, were summarized as farm-level means; categorical cow-level outcomes, such as lameness, low body condition score, lesions, hygiene status, and CMT status, were summarized as farm-level prevalences. Group summaries of these prevalence outcomes represent the mean of the within-farm proportions, rather than a pooled prevalence calculated across all assessed cows.

#### Compliance scoring

Cow comfort compliance was calculated from 15 cow comfort recommendation items, using the scoring structure previously applied in Kenyan smallholder dairy research (Kariuki et al., 2023)(i.e., weighting recommendations by whether they required major or minor effort and whether they were fully, partially or not implemented). Each recommendation item k had two components at follow-up: a recommendation weight coded as none (0), minor (1) and major (2); and an implementation quality score, coded as not done (0), partly done (1) and well done (2). For each recommendation item, a weighted item score (0 – 4) was computed as the product of recommendation weight and implementation quality:

$$S_{ik}=recom_{ik}\times comply_{ik},S_{ik}\in \left\{0,1,2,4\right\}$$

The score gives greater weight to recommendations classified as requiring major effort. A partially implemented major recommendation and a fully implemented minor recommendation each contribute two points to the numerator. This equivalence is a feature of the scoring convention and does not imply equivalent effects on cow welfare. Recommendation eligibility and effort weights were established when the initial recommendations were issued and held fixed during follow-up; implementation status was reassessed at each visit. Since every $i^{th}$ farm received specific recommendations depending on its baseline cow comfort status (different number and types of recommendations), its compliance was normalized to the maximum achievable score. The maximum score was:

$$S_{i}^{max}=2\sum_{k}recom_{ik}$$

and the total achieved score was computed as follows:

$$S_{i}^{achieved}=\sum_{k}S_{ik}$$

Then, compliance percentage score for the $i^{th}$ farm was:

$$C_{i}=100\;\times \;\frac{S_{i}^{achieved}}{S_{i}^{max}},\text{computed}\;\text{only}\;\text{when}\;S_{i}^{max}>0).$$

For example, a farm with one major recommendation implemented partially and one minor recommendation implemented fully receives 4 of a maximum 6 points, giving a compliance score of 66.7%. The score expresses implementation relative to the farm’s own recommendation package; it does not represent the percentage of recommendations completed fully. This scoring approach has been used previously in Kenyan smallholder dairy research (Kariuki et al., 2023), but has not undergone formal validation as a measure of welfare benefit.

#### Reinforcement assignment and compliance

The primary analysis estimated the difference in mean endline compliance between reinforcement assignment groups among intervention farms with observed and defined compliance scores (i.e., $S_{i}^{max}>0)$:

$$C_{i,end}=\;\alpha +\;\beta R_{i}+X_{i}^{T}\gamma +\varepsilon_{i},$$

Where $C_{i,end}$ is the endline compliance (%), $R_{i}$ is the reinforcement assignment indicator, and $X_{i}$ comprises a small set of pre-specified, pre-randomization baseline covariates included solely for precision. The covariates selected a priori were baseline indicators of farm capacity (e.g., herd size) and baseline cow comfort/stall condition status (summarized as a stall-deficit measure), together with fixed effects for geographical region.

Ordinary least squares regression was used to estimate percentage-point differences in compliance, with robust standard errors. A fully interacted model, including reinforcement-by-baseline-covariate interactions, was used as a sensitivity analysis, and its marginal reinforcement contrast was reported. Endline cow-comfort compliance was specified as the primary outcome; milk yield and lameness were treated as key secondary outcomes, while the remaining welfare, health, and stall-condition endpoints were interpreted as exploratory. Statistical tests were two-sided, with p < 0.05 used as the threshold for statistical significance. P-values and 95% confidence intervals were not adjusted for multiple comparisons; findings for exploratory outcomes were interpreted cautiously, with emphasis on effect estimates and their confidence intervals.

#### Reinforcement assignment and animal outcomes

Differences in endline welfare outcomes and milk yield between reinforcement assignment groups were estimated using analysis of covariance (ANCOVA) models with heteroskedasticity-robust standard errors following Greene (2003): $Y_{i,end}=\alpha +\;\beta R_{i}+\delta Y_{i,base}+X_{i}^{T}\gamma +\varepsilon_{i},$ where $Y_{i,end}$ and $Y_{i,base}$ represent each welfare/productivity endpoint and baseline, respectively. For cow-level continuous normally distributed outcomes (e.g., milk yield), a farm-level mean was calculated, with inference clustered at the farm level to reflect the unit of randomization. Categorical outcomes measured at the cow level (e.g., lameness scores, body condition scores, and neck/hock/carpus lesion scores) were summarized at the farm level using prevalence rather than farm-level mean scores, according to the outcome-specific thresholds described below.

#### The association between achieved compliance and welfare/productivity outcomes

Given that compliance was a post-randomization behaviour, estimating its association with welfare or productivity requires addressing confounding: farms with higher compliance may differ systematically in resources, labor availability, baseline comfort status, or management capacity. We therefore used inverse-probability-weighted models to estimate associations between achieved compliance and endline outcomes among farms with a defined compliance score (Cole & Hernan, 2008; Hernán & Robins, 2017), subject to $S_{i}^{max}>0$.

Following Kariuki et al. (2023), this study defined a binary compliance exposure $A_{i}$ using a weighted compliance score of ≥40% to identify the higher-compliance category ($A_{i}$ = 1 denoting high compliance if farm i achieved a score ≥40%; otherwise, $A_{i}$ = 0). Kariuki et al. used the same scoring construction and described a natural break near 40% in their study population. The threshold was adopted to support comparison with that earlier work. It is an operational classification and has not been validated as a minimum level of implementation required to improve welfare.

This was followed by estimation of the probability of observing each farm’s realized compliance level, conditional on baseline covariates $L_{i}$ (e.g., herd size, baseline stall-condition status and management indicators). Stabilized inverse probability weights for achieved compliance were computed as follows: $SW_{i}=\frac{P(A_{i})}{P(A_{i}|L_{i})}$. Additionally, to address selection due to loss to follow-up (attrition), stabilized censoring weights were estimated, when necessary, $SW_{i}^{C}=\frac{P(C_{i}=0)}{P(C_{i}=0|L_{i})}$, where $C_{i}$ indicates censoring/attrition.

The final analysis weights were $W_{i}=SW_{i}\times SW_{i}^{C}$. The combined weights were used to adjust the compliance–outcome comparison for measured predictors included in the compliance and observation models. Weighted outcome models were fitted with robust standard errors. These analyses were interpreted as post-randomization associations because achieved compliance was not randomized. For milk yield, $Y_{i,\;end}$ was analyzed on a continuous scale. For animal-based welfare endpoints related to cow comfort (lameness, BCS categories, and lesions), outcomes were represented as within-farm prevalences and analyzed using weighted generalized linear models to estimate marginal contrasts (Dohoo et al., 2014).

Specifically, for the cow-level continuous outcomes, such as milk yield, cow observations were averaged within farm before analysis. For cow-level categorical or ordinal welfare outcomes, scores were converted into binary indicators and then summarized as within-farm prevalences. Lameness was defined as a locomotion score ≥2, low body condition as a body condition score <2.5, and lesions as the presence of any lesion. These farm-level prevalence outcomes were analysed using weighted linear models to estimate differences in mean within-farm prevalence, expressed in percentage points, with fractional logit models used as sensitivity analyses for bounded outcomes.

#### Paired item-level implementation

Paired midline-to-endline analyses described changes in implementation among reinforcement recipients observed at both visits. Each item-specific analysis was restricted to farms recorded as recommended that item at midline and assessed at both visits. Any implementation was coded as 1 for partial or full implementation and 0 for no implementation. We calculated the implementation proportion at each visit and the endline-minus-midline difference in percentage points. Continuity-corrected McNemar tests assessed changes in the paired binary outcomes. These analyses described changes within reinforced farms and did not compare reinforcement with no reinforcement.

#### Diagnostics and sensitivity analyses

This study evaluated four diagnostic domains recommended for inverse probability weighting analyses (Cole & Hernan, 2008; Hernán & Robins, 2017). First, to assess whether the two compliance strata were represented across covariate patterns, we inspected the distribution of predicted probabilities $P(A_{i}|L_{i})$ for overlaps. Second, we summarized the distribution of stabilized weights (mean, SD and upper percentiles) and implemented weight truncation sensitivity analysis to check and reduce undue influence of extreme weights. Third, we evaluated covariate balance after weighting by comparing standardized mean differences for key baseline covariates between high- and low-compliance farms before and after weighting. Lastly, we compared estimates using stabilized compliance weights alone with estimates using combined compliance and censoring weights truncated at the 1st and 99th percentiles.

## Results

The 318 smallholder dairy farms in the study had mostly (60%) male household heads. At enrollment, the mean number of lactating cows assessed per farm was 2.04, with a median of 2 cows. Farm-level mean of cow liveweight averaged 448.2 kg (95% CI 438.5–457.9) at baseline and was 456.2 kg in intervention farms and 432.2 kg in control farms. Mean milk yield was 7.49 kg/day overall, 7.53 kg/day in intervention farms and 7.40 kg/day in control farms. All other baseline farm descriptors and farmer-reported management practices are summarized fully in Table 2, including arm-specific summaries.

**Table 2:** Baseline farm, farmer and stall characteristics.

Farm parameter	Levels	Overall (n = 318)	Intervention (n = 212)	Control (n = 106)
Male household head (%)		60.06	58.02	64.15
Age of household head, years, mean		45.83	46.21	45.01
Number of lactating cows, median (IQR)		2 (1–3)	2 (1–3)	2 (1–2)
Floor type	Concrete	51.6	48.6	57.5
	Dirt	46.5	49.5	40.6
	Other	1.9	1.9	1.9
Lying area manure removal frequency	> Once a day	29.6	30.7	27.4
	Once a day	53.5	52.4	55.7
	Every other day	2.5	3.3	0.9
	Once a week	6.0	6.1	5.7
	Twice a week	4.4	3.8	5.7
	< Once a week	4.1	3.8	4.7
Alleyway manure removal frequency	> Once a day	32.7	34.9	28.3
	Once a day	50.6	48.1	55.7
	Every other day	1.3	1.9	0.0
	Once a week	6.6	7.1	5.7
	Twice a week	4.1	3.3	5.7
	< Once a week	4.7	4.7	4.7
Stall hardness rating	Poor	17.6	19.3	14.2
	Fair	51.9	51.9	51.9
	Good	27.0	25.5	30.2
	Very good	3.1	2.8	3.8
	Excellent	0.3	0.5	0.0
Stall wetness rating	Poor	26.1	29.7	18.9
	Fair	51.9	47.6	60.4
	Good	19.5	20.8	17.0
	Very Good	2.5	1.9	3.8

### Baseline cow comfort and stall environment

Self-reported stall infrastructure and management indicated substantial conditions that needed improvement. Concrete was the most common lying-surface type (51.6%), followed by dirt (46.5%). Bedding use in the lying area was reported by 36.8% of the farms, and 54.1% of the farms had stalls with a lumpy lying surface. In direct stall-condition assessments aggregated to the farm level, based on the knee-impact and knee-wetness tests, the mean within-farm proportions classified as inadequate were 74.2% for stall hardness and 79.4% for stall wetness.

Stall design inadequacies were also common: inadequate stall width (mean prevalence 59.7%) and inadequate stall length (56.8%) were frequent, while inadequate body length (95.1%), inadequate/absent neck rail (96.3%) and inadequate/absent brisket board (99.8%) were found in almost all farms. Table 3 summarizes all assessed cow comfort and related cow health indicators at baseline.

**Table 3:** Baseline prevalence of cow comfort and health indicators^1^.

Cow comfort parameter	Overall, n = 318 (%)		Intervention, n = 212 (%)		Control, n = 106 (%)	
	Prev	95%CI	Prev	95%CI	Prev	95%CI
Animal-based outcomes						
Dirty udder	8.42	5.78 to 11.07	8.70	5.58 to 11.83	7.86	2.94 to 12.78
Dirty legs	34.96	30.25 to 39.67	35.35	29.63 to 41.08	34.17	25.86 to 42.47
Neck lesions	10.42	7.53 to 13.32	11.93	8.14 to 15.71	7.42	3.19 to 11.65
Hock lesions	13.10	9.73 to 16.47	14.96	10.56 to 19.36	9.39	4.46 to 14.31
Carpus lesions	5.83	3.57 to 8.08	4.32	2.02 to 6.63	8.84	3.92 to 13.76
Body condition score (<2.5)	39.02	34.34 to 43.70	42.4	36.68 to 48.12	32.26	24.23 to 40.29
Lameness score (≥2)	48.6	43.63 to 53.57	46.38	40.26 to 52.51	53.03	44.59 to 61.48
CMT score ≥1 in at least one-quarter	51.18	46.53 to 55.84	50.52	44.82 to 56.22	52.52	44.41 to 60.62
CMT score ≥2 in at least one-quarter	28.85	24.68 to 33.02	27.72	22.67 to 32.77	31.1	23.71 to 38.49
Cow hits wood	23.81	19.54 to 28.07	24.48	19.24 to 29.72	22.45	15.10 to 29.81
Odd stall use	75.16	70.29 to 80.02	69.34	62.75 to 75.94	84.47	77.83 to 91.10
Stall measurements and conditions						
Inadequate stall width	59.74	54.74 to 64.73	58.35	52.16 to 64.54	62.52	54.05 to 70.98
Inadequate stall length	56.77	51.76 to 61.77	56.12	49.93 to 62.30	58.07	49.52 to 66.61
Inadequate body length	95.06	92.93 to 97.19	93.87	90.93 to 96.81	97.45	95.01 to 99.90
Inadequate/no brisket board	99.76	99.42 to 100	99.65	99.13 to 100	100	-
Inadequate/no neck rail	96.34	94.52 to 98.15	96.72	94.64 to 98.80	95.57	92.04 to 99.10
Inadequate lunge space	14.2	10.60 to 17.81	18.98	13.95 to 24.01	4.65	1.35 to 7.95
Inadequate leg space	17.22	13.21 to 21.22	16.78	11.84 to 21.72	18.08	11.21 to 24.95
Hard stall base (Knee impact)	74.23	69.53 to 78.93	74.47	68.72 to 80.21	73.77	65.56 to 81.99
Wet stall base (Knee wetness)	79.44	75.10 to 83.78	81.01	75.83 to 86.19	76.29	68.41 to 84.17
Dirty stalls	31.16	26.20 to 36.11	33.53	27.36 to 39.70	26.42	18.16 to 34.67
Bedding used in lying area	36.8	31.7 to 42.2	37.70	31.50 to 44.40	34.90	26.50 to 44.40
Lumpy floor	54.1	48.6 to 59.5	52.80	46.10 to 59.40	56.60	47.10 to 65.60
Sharp objects present	28.6	23.9 to 33.8	30.70	24.80 to 37.20	24.50	17.30 to 33.50
Roof appropriate	90.6	86.9 to 93.3	89.60	84.80 to 93.00	92.50	85.80 to 96.10

Animal-based entries are means of within-farm prevalences. Stall entries are summarized according to the assessed stall denominator. Farm-level binary characteristics are reported as percentages of farms. “Inadequate” includes marginal and not-good ratings.

### Baseline welfare and health indicators linked to cow comfort

The animal-based welfare indicators related to cow comfort summarized as within-farm prevalences (Table 3) suggested a high baseline burden of compromised comfort. The mean within-farm prevalence of lameness (scores ≥2) was 48.6%, and the prevalence of low body condition scores (BCS <2.5) was higher in farms in the intervention arm compared to those in the control arm (42.4%vs 32.3%, respectively). Mean prevalence of lesions was 10.4% for neck lesions, 13.1% for hock lesions and 5.8% for carpus lesions. At baseline, the mean within-farm prevalence of cows with positive CMT scores ≥1 in at least one-quarter was 51.2%; the corresponding prevalence for scores ≥2 was 28.9%.

### Attrition and follow-up participation

Overall endline completion was 86.8% (n = 276), with 13.2% of farms lost to follow-up. Endline assessments were completed for 185 of 212 intervention farms and 91 of 106 control farms. Within the intervention arm, 97 of 106 farms assigned to reinforcement (91.5%) and 88 of 106 farms assigned to no reinforcement (83.0%) were assessed at endline, an absolute difference of 8.5 percentage points. The corresponding Pearson χ² statistic was 3.438 (1° of freedom; p = 0.064). Among the 85 farms that received reinforcement, 83 completed endline assessment (97.6%).

To evaluate whether attrition plausibly introduced selection bias, retained vs. attrited farms were compared on key baseline production and welfare/comfort measures using Wilcoxon rank-sum tests and χ² tests for continuous and categorical variables, respectively. Attrition was not detectably associated with baseline milk yield, cow weight, CMT positivity, or low BCS. The only exceptions were bedding use and baseline wet floor, which differed marginally between completers and attriters.

### Item-level implementation of cow comfort recommendations

Implementation of recommendations across the 15 cow comfort items was summarized in Table 4. Out of 185 intervention farms evaluated at endline, the highest prevalences of farm-specific recommendations provided were on stall components: 87% on body length recommendations, 86.5% on neck rail recommendations and 78.4% on brisket board recommendations. In contrast, roof-water (5.9%) and surface-water (5.4%) management received the least recommendations. For each of the cow comfort items, Table 4 details (i) the proportion of intervention farms that received the recommendation, and, conditional on being recommended, (ii) the proportion that achieved either full or partial implementation.

**Table 4:** Item-level implementation of cow-comfort recommendations among intervention farms assessed at endline.

Recommendation	Farms recorded as recommended the item, n/N (%)	Any implementation, n/n recommended (%)	Full implementation, n/n recommended (%)
Roof water	11/185 (5.9)	2/11 (18.2)	1/11 (9.1)
Surface water	10/185 (5.4)	2/10 (20.0)	2/10 (20.0)
Floor softness	142/185 (76.8)	73/142 (51.4)	36/142 (25.4)
Floor dryness	71/185 (38.4)	30/71 (42.3)	15/71 (21.1)
Floor evenness	79/185 (42.7)	44/79 (55.7)	25/79 (31.6)
Total width	105/185 (56.8)	33/105 (31.4)	30/105 (28.6)
Total length	16/185 (8.6)	7/16 (43.8)	6/16 (37.5)
Body length	161/185 (87.0)	46/161 (28.6)	33/161 (20.5)
Forward/side lunge	11/185 (5.9)	6/11 (54.5)	4/11 (36.4)
Leg space	22/185 (11.9)	12/22 (54.5)	10/22 (45.5)
Brisket board	145/185 (78.4)	14/145 (9.7)	7/145 (4.8)
Neck rail	160/185 (86.5)	44/160 (27.5)	23/160 (14.4)
Clean alley	51/185 (27.6)	22/51 (43.1)	10/51 (19.6)
Sharps fixed	18/185 (9.7)	6/18 (33.3)	4/18 (22.2)
Round pole on feeding area	19/185 (10.3)	12/19 (63.2)	4/19 (21.1)

Any implementation includes partial or full implementation. Full implementation is a subset of any implementation. Recommendation frequencies use the 185 intervention farms assessed at endline as the denominator. Implementation percentages use the number of farms recorded as recommended the corresponding item.

In the endline item-specific summaries, full implementation ranged from 4.8% for brisket-board recommendations to 45.5% for leg-space recommendations. Any implementation, comprising partial or full implementation, was reported for 63.2% of farms recorded as recommended a round pole over the feeding area, 55.7% for floor evenness, 54.5% for forward/side lunge space, and 51.4% for floor softness. Corresponding proportions were lower for brisket boards (9.7%), neck rails (27.5%), and body length (28.6%). Full implementation was included within, rather than additional to, these any-implementation percentages.

The paired cohort comprised 83 reinforcement recipients observed at midline and endline, with item-specific samples ranging from 6 to 72 farms (Table 5). Any implementation increased from 7/22 (31.8%) to 18/22 (81.8%) for total stall length, from 3/10 (30.0%) to 10/10 (100.0%) for forward/side lunge space, and from 6/21 (28.6%) to 16/21 (76.2%) for leg space. The corresponding increases were 50.0, 70.0 and 47.6 percentage points, with continuity-corrected McNemar p-values of 0.006, 0.023 and 0.016, respectively. These exploratory comparisons describe within-farm changes among reinforcement recipients.

**Table 5:** Paired changes in item-level implementation between midline and endline among farms receiving reinforcement.

Recommendation	Paired farms, n	Midline any implementation, n (%)	Endline any implementation, n (%)	Change, percentage points	McNemar p-value
Roof water	6	4 (66.7)	3 (50.0)	−16.7	>0.999
Surface water	7	3 (42.9)	5 (71.4)	28.6	0.480
Floor softness	62	25 (40.3)	35 (56.5)	16.1	**0.034**
Floor dryness	31	18 (58.1)	23 (74.2)	16.1	0.228
Floor evenness	38	18 (47.4)	26 (68.4)	21.1	**0.080**
Total width	49	14 (28.6)	22 (44.9)	16.3	**0.043**
Total length	22	7 (31.8)	18 (81.8)	50.0	**0.006**
Body length	62	9 (14.5)	19 (30.6)	16.1	**0.016**
Forward/side lunge	10	3 (30.0)	10 (100.0)	70.0	**0.023**
Leg space	21	6 (28.6)	16 (76.2)	47.6	**0.016**
Brisket board	68	5 (7.4)	15 (22.1)	14.7	**0.004**
Neck rail	72	12 (16.7)	24 (33.3)	16.7	**0.003**
Clean alley	28	16 (57.1)	20 (71.4)	14.3	0.386
Sharps fixed	14	7 (50.0)	10 (71.4)	21.4	0.248
Round pole on feeding area	13	8 (61.5)	11 (84.6)	23.1	0.248

The overall paired cohort comprised 83 farms observed at midline and endline. Each analysis was restricted to farms recorded as recommended the corresponding item at midline, giving item-specific denominators of 6–72 farms. Any implementation comprises partial or full implementation. The same item-specific denominator is used at both visits. Changes are endline minus midline percentages, calculated before rounding. P-values are from continuity-corrected McNemar tests and were not adjusted for multiple comparisons.

### Reinforcement assignment and compliance

Overall, among intervention farms with a defined score, the endline compliance percentages were bounded and strongly right-skewed, with substantial proportion of farms having zero compliance (median 16.7%, IQR 0–50; mean 30.4%, n = 182). Similarly, for the intervention farms assessed at midline during the reinforcement visits, compliance score distributions were right-skewed (median 14.3%, IQR 0–42.9; mean 25.3%, n = 85). At midline, 29.3% (95% CI 20.3–40.1) reinforcement-assigned farms with a defined cow-comfort compliance score met the ≥40% high-compliance threshold; while at endline, 35.7% met this high-compliance threshold (95% CI 29.0–43.0).

As reported in Table 6, the farms that were assigned to reinforcement had higher mean endline compliance (35.8%) compared to the farms that were not assigned to reinforcement (24.6%). A nonparametric comparison of the compliance distributions (Wilcoxon rank-sum) indicated statistically significant evidence of a shift in the distribution towards a higher compliance under reinforcement (p = 0.028). In the unadjusted OLS model, assignment to reinforcement was associated with a statistically significant 11.2 percentage-point higher endline cow-comfort compliance score (p = 0.025). After adjusting for the baseline covariates pre-specified in the analytical framework (herd size, stall condition and region fixed effects), reinforcement was associated with a 12.0 percentage-point higher endline compliance (p = 0.012). A Lin-style specification allowing treatment-by-covariate interactions yielded a similar marginal contrast from predictive margins: 12.1 percentage points (p = 0.010).

**Table 6:** Effect of reinforcement on endline cow-comfort compliance (intervention arm).

Endline compliance distribution					
Reinforcement assignment	n	Mean (%)	SD	Median (%)	IQR (%)
No reinforcement	88	24.6	31.9	10.0	0–46.4
Reinforcement	94	35.8	35.0	25.0	0–66.7
Overall	182	30.4	33.9	16.7	0–50.0
*Differences by randomized reinforcement assignment among farms with defined endline compliance scores*					
OLS Models	Estimate	SE	95% CI	p-value	n
Unadjusted difference in means	11.2	4.9	1.4 to 21.0	0.025	182
Adjusted difference in means	12.0	4.7	2.7 to 21.3	0.012	182
Lin-style (with interactions); marginal contrast	12.1	4.7	2.9 to 21.4	0.010	182

*Of the 185 intervention farms assessed at endline, 182 had defined endline compliance scores: 94 assigned to reinforcement and 88 assigned to no reinforcement. Three reinforcement-assigned farms were excluded from this analysis: two had missing endline compliance scores and one had no eligible recommendations. OLS, ordinary least squares; SE, standard error; SD, standard deviation; CI, confidence interval; IQR, interquartile range.

### Differences in productivity and welfare outcomes by reinforcement assignment

Among intervention farms with the required outcome data, ANCOVA models compared endline outcomes between reinforcement assignment groups, adjusting for the baseline value of the outcome and geographical region, with standard errors clustered at the farm level. For the prevalence-type outcomes summarized as farm-level prevalences, such as the animal-welfare and health parameters, the primary estimand was reported as an OLS difference in mean within-farm prevalence, expressed in percentage points. Fractional logit specification was also estimated for each prevalence endpoint as a sensitivity analysis with model-based adjusted prevalences by reinforcement arm and marginal contrasts.

Table 7 presents outcome-specific differences by randomized reinforcement assignment. Among farms included in the outcome analyses, reinforcement assignment was associated with higher endline milk yield – the estimated mean difference was +1.33 kg/day (95% CI 0.32 - 2.35; p = 0.011). Across both specifications, reinforcement assignment was associated with a lower endline prevalence of lameness. The OLS ANCOVA difference was −19.75 percentage points (95% CI −33.55 to −5.96; p = 0.005), and the fractional-logit marginal difference was −19.58 percentage points (95% CI −33.06 to −6.10; p = 0.004), consistent in magnitude and direction with the OLS contrast.

**Table 7:** Differences in endline outcomes by randomized reinforcement assignment among farms with observed outcomes.

Outcome	n	ANCOVA OLS	Fractional Logit (margins)						
		Mean or prevalence differences	95% C. I	p value	Adjusted prevalence with reinforcement	Adjusted prevalence without reinforcement	Δ diff	95% C. I	p value
Mean milk yield (kg/day)	183	1.33	0.32 to 2.35	**0.011**					
Lameness, locomotion score (≥2)	185	−19.75	−33.55 to −5.96	**0.005**	31.8	51.4	−19.58	−33.06 to −6.10	**0.004**
Low BCS (<2.5)	185	1.67	−10.55 to 13.89	0.787	32.3	30.5	1.8	−10.09 to 13.69	0.767
Any neck lesion	185	−3.57	−10.94 to 3.80	0.340	9.1	13.2	−4.06	−11.56 to 3.43	0.288
Any hock lesion	185	−5.41	−13.79 to 2.97	0.205	9.0	14.3	−5.37	−13.95 to 3.20	0.219
Any carpus lesion	185	0.44	−3.64 to 4.52	0.832	2.5	1.5	0.92	−3.38 to 5.23	0.675
CMT score ≥1 in at least one-quarter	185	−8.74	−20.56 to 3.07	0.146	47.9	56.6	−8.76	−20.20 to 2.68	0.133
CMT score ≥2 in at least one-quarter	185	−6.44	−17.73 to 4.84	0.261	31.4	37.6	−6.2	−17.24 to 4.85	0.272
Mastitis in the last month	185	2.25	−1.98 to 6.47	0.295	5.1	2.9	2.29	−1.87 to 6.45	0.281
Cow hits wood when standing up	185	1.11	−2.71 to 4.93	0.566	3.1	2.0	1.1	−2.62 to 4.83	0.562
Cow lies down outside stall	185	0.22	−12.97 to 13.40	0.974	48.2	47.8	0.45	−12.25 to 13.15	0.945
Odd stall use	145	−11.41	−28.02 to 5.20	0.177	35.6	46.9	−11.29	−27.15 to 4.57	0.163
Dirty udders	185	0.14	−4.13 to 4.41	0.948	3.7	4.0	−0.29	−4.45 to 3.86	0.891
Dirty legs	185	0.41	−8.43 to 9.24	0.928	16.9	16.8	0.06	−8.20 to 8.32	0.988
Dirty stalls	185	0.92	−7.15 to 8.99	0.822	9.9	9.2	0.78	−7.03 to 8.58	0.846
*Stall-condition measures related to components of the compliance score*									
Hard stall floor	185	−7.43	−19.19 to 4.32	0.214	53.6	61.4	−7.82	−19.10 to 3.46	0.174
Wet stall floor	185	−9.5	−23.65 to 4.65	0.187	25.8	35.2	−9.4	−23.16 to 4.36	0.181
Inadequate stall width	185	−16.72	−30.23 to −3.20	**0.016**	32.5	49.3	−16.79	−29.93 to −3.64	**0.012**
Inadequate stall total length	185	3.12	−4.25 to 10.49	0.405	7.1	3.5	3.58	−4.11 to 11.27	0.362
Inadequate stall body length	185	−18.82	−31.98 to −5.65	**0.005**	60.1	79.1	−18.96	−31.74 to −6.19	**0.004**
Inadequate stall brisket board	185	−5.37	−17.89 to 7.15	0.398	*	*	*	*	
Inadequate stall neck rail	185	−17.42	−29.80 to −5.03	**0.006**	65.6	83.3	−17.65	−29.70 to −5.61	**0.004**
Inadequate stall lunge space	185	−4.09	−10.46 to 2.28	0.207	2.0	6.0	−4.02	−10.49 to 2.46	0.224
Inadequate stall leg space	185	0.81	−6.75 to 8.36	0.834	6.9	6.1	0.81	−6.55 to 8.17	0.830

* The fractional-logit model for inadequate brisket-board status did not converge; adjusted prevalences and marginal contrasts are therefore not reported. Milk-yield estimates are mean differences in kg/day. Estimates for prevalence outcomes are percentage-point differences. Fractional-logit contrasts were calculated from model-based adjusted prevalences. ANCOVA, analysis of covariance; OLS, ordinary least squares; CI, confidence interval; BCS, body condition score; CMT, California Mastitis Test.

With respect to cow comfort related stall conditions, several structure deficits showed lower endline prevalence for farms assigned to the midterm reinforcement. In OLS ANCOVA, inadequate stall width was −16.7 percentage points (95% CI −30.2 – −3.2; p = 0.016), inadequate body length was −18.82 percentage points (95% CI −31.98 – −5.65; p = 0.005), and inadequate neck rail was −17.4 percentage points (95% CI −29.8 – −5.0; p = 0.006), with fractional logit margins yielding closely similar marginal contrasts for width (p = 0.012), body length (p = 0.004), and neck rail (p = 0.004). For the remaining estimable contrasts, the 95% confidence intervals included zero.

### The association between achieving high compliance and welfare and productivity

The final objective of this study was to estimate whether achieving high cow comfort compliance scores was associated with measurable productivity and welfare differences. Table 8 presents weighted associations between achieved compliance and endline productivity and welfare outcomes. Higher compliance was associated with an estimated 1.00 kg/day higher milk yield, but the confidence interval included both a small negative difference and a positive difference (95% CI −0.27 to 2.27; p = 0.121).

**Table 8:** Weighted associations between higher compliance and endline outcomes.

Outcome	**n**	**Weighted ANCOVA (OLS)**				Fractional Logit				
		Prevalence difference	95% C I	p value		Adjusted prevalence with compliance ≥40%	Adjusted prevalence with compliance <40%, %	Δdiff	95% C I	p value
Mean milk yield (kg/day)	180	1.0	−0.27 to 2.27	0.121						
Lameness, locomotion scores (≥2)	182	−49.39	−60.64 to −38.15	**<0.001**		8.2	57.6	−49.45	−60.08 to −38.83	**<0.001**
Low Body Condition Score (<2.5)	182	−2.04	−14.65 to 10.58	0.750		30	32.1	−2.06	−14.31 to 10.19	0.742
Any neck lesion	182	1.2	−7.47 to 9.88	0.785		12.3	11.1	1.18	−7.15 to 9.51	0.781
Any hock lesion	182	2.49	−7.85 to 12.83	0.635		14.3	11.8	2.44	−7.83 to 12.72	0.641
Any carpus lesion	182	1.66	−2.34 to 5.67	0.414		2.3	1.1	1.25	−2.19 to 4.69	0.475
CMT score ≥1 in at least one-quarter	182	1.64	−11.20 to 14.47	0.801		52.5	50.8	1.66	−10.87 to 14.18	0.796
CMT score ≥2 in at least one-quarter	182	−1.17	−12.66 to 10.31	0.840		32.6	34	−1.33	−12.55 to 9.89	0.816
Mastitis in the last month	182	3.34	−2.30 to 8.97	0.244		6.3	3	3.29	−2.03 to 8.61	0.225
Dirty udders	182	−2.33	−6.43 to 1.78	0.265		2.2	4.9	−2.61	−6.60 to 1.37	0.199
Cow hits wood when standing up	182	0.71	−4.07 to 5.49	0.770		3.1	2.4	0.7	−4.01 to 5.41	0.770
Cow lies down outside stall	182	−15.79	−29.09 to −2.50	**0.020**		37.6	53.4	−15.88	−28.64 to −3.11	**0.015**
Odd stall use	142	−20.9	−36.40 to −5.41	**0.009**		25.7	46.6	−20.94	−35.86 to −6.01	**0.006**
Dirty legs	182	4.85	−5.26 to 14.96	0.345		20.8	15.5	5.29	−4.59 to 15.17	0.294
Dirty stalls	182	−5.05	−12.28 to 2.17	0.169		5.9	11	−5.14	−12.18 to 1.89	0.152
*Stall-condition measures related to components of the compliance score*										
Hard stall floor	182	−37.06	−49.97 to −24.16	**<0.001**		33.5	70.2	−36.77	−49.04 to −24.50	**<0.001**
Wet stall floor	182	−19.69	−33.15 to −6.23	**0.004**		17.4	37.1	−19.65	−32.64 to −6.66	**0.003**
Inadequate stall width	182	−45.59	−57.64 to −33.53	**<0.001**		8.5	55.6	−47.18	−58.47 to −35.90	**<0.001**
Inadequate stall total length	182	−6.39	−12.03 to −0.75	**0.027**		1.2	7.6	−6.4	−11.49 to −1.32	**0.014**
Inadequate stall body length	182	−62.34	−75.30 to −49.39	**<0.001**		30.4	92.7	−62.24	−74.80 to −49.68	**<0.001**
Inadequate stall brisket board	182	−31.7	−45.79 to −17.61	**<0.001**		54	86.2	*	*	
Inadequate stall neck rail	182	−47.88	−61.57 to −34.19	**<0.001**		45.2	92.7	−47.49	−60.59 to −34.38	**<0.001**
Inadequate stall lunge space	182	−0.56	−6.16 to 5.04	0.844		3.5	4	−0.58	−5.62 to 4.46	0.822
Inadequate stall leg space	182	−7.01	−14.19 to 0.17	0.056		3.1	9.5	−6.37	−13.65 to 0.92	0.087

N denotes the number of farms included in each outcome-specific analysis. * The fractional-logit model for inadequate brisket board did not converge; Compliance was measured after randomization; these comparisons are interpreted as associations.

For the animal-based welfare related outcomes, lameness had the largest weighted contrasts. Higher compliance was associated with a markedly lower endline lameness prevalence: the weighted OLS difference in mean within-farm prevalence was −49.39 percentage point (95% CI −60.64 to −38.15; p < 0.001), while the fractional logit margin yielded a similar marginal difference (−49.45 pp, 95% CI −60.08 to −38.83; p < 0.001).

High compliance was associated with substantially lower prevalence of several stall-structure deficits. The weighted OLS contrasts indicated lower endline prevalences for stall-floor hardness assessed using the knee-impact test (−37.06 pp; p < 0.001), stall-floor dampness assessed using knee wetness test (−19.69 pp; p = 0.004), inadequate total width (−45.59 pp; p < 0.001), inadequate total length (−6.39 pp; p = 0.027), inadequate body length (−62.34 pp; p < 0.001), and inadequate or missing neck rail (−47.88 pp; p < 0.001). The fractional logit margins were similar in direction and magnitude for these cow comfort stall parameters. Furthermore, lying outside the stall differed by −15.79 pp (p = 0.020), and odd stall use differed by −20.90 pp (p = 0.009). Several stall-condition outcomes describe conditions directly targeted by recommendations contributing to the compliance score. Their associations with compliance partly reflect this overlap and should be interpreted as implementation findings. The associations involving lying outside the stall and odd stall use remain observational and share the confounding and temporal limitations of the other achieved-compliance analyses.

### Diagnostic checks and sensitivity analysis

As recommended for inverse-probability-weighted analyses, key diagnostic domains were evaluated. First, upon assessment of the overlap in the predicted probability of high compliance from the denominator model to evaluate positivity, predicted probabilities ranged from 0.101 to 0.720 (median 0.343, IQR 0.266–0.438; mean 0.357, SD 0.137). These summaries did not indicate predicted probabilities close to zero or one; assessment of overlap also requires examining their distributions within each compliance category.

Second, inspecting the stabilized weight distributions ($SW_{i}^{A}$) indicated that the compliance weights were centered near one with a limited upper tail (mean 1.002, SD 0.207, max 2.032), and censoring weights ($SW_{i}^{C}$) for endline observation were similarly well-behaved (mean 1.000, SD 0.059, max 1.239). The combined weights, $W_{i}=SW_{i}^{A}\times SW_{i}^{C}$ remained stable (mean ≈ 1.001, max 1.846), and truncation at the 1st/99th percentiles bounded the range of $W_{i}$ to 0.680 – 1.775.

Third, covariate balance across compliance strata was assessed by comparing baseline covariate means before and after weighting. Before weighting, some of the baseline characteristics differed between high compliance and low compliance farms; after weighting, by $W_{i}$, mean differences were attenuated and key baseline covariates aligned closely between strata (e.g., herd size, baseline milk yield, baseline stall conditions), indicating improved balance in the measured covariates examined.

Table 9 compares estimates using stabilized compliance weights alone with estimates using truncated combined compliance and censoring weights. The estimates were similar for the outcomes shown. Because both censoring adjustment and truncation changed between specifications, this comparison does not isolate the effect of censoring adjustment.

**Table 9:** Sensitivity of weighted compliance associations to the weight specification.

Outcome	**Stabilized IPTW for compliance**					**Truncated stabilized combined weight**			
	**Estimate**	**95% Confidence interval**		**p-value**	**Estimate**		**95% Confidence interval**		**p-value**
		**Lower**	**Upper**				**Lower**	**Upper**	
Milk yield (kg/day)	0.960	−0.30	2.22	0.134	0.999		−0.27	2.27	0.121
Lameness, locomotion score	−50.07	−61.29	−38.85	<0.001	−49.39		−60.64	−38.15	<0.001
Inadequate stall width	−46.06	−58.22	−33.89	<0.001	−45.59		−57.64	−33.53	<0.001
Hard stall floor	−37.54	−50.59	−24.50	<0.001	−37.06		−49.97	−24.16	<0.001
CMT positive prevalence	1.44	−11.41	14.29	0.825	1.64		−11.20	14.47	0.801

Estimates compare compliance ≥40% with compliance <40%. Milk-yield differences are expressed in kg/day; all other differences and confidence limits are expressed in percentage points. The first specification uses stabilized compliance weights; the second uses combined compliance and censoring weights truncated at the 1st and 99th percentiles. CI, confidence interval; IPTW, inverse-probability-of-treatment weighting.

## Discussion

The main objective of this study was to assess whether assignment to reinforcement increased endline compliance and whether achieved compliance was associated with endline welfare outcomes and milk yield. Farms assigned to midterm reinforcement had higher endline compliance than farms assigned to receive recommendations alone. Implementation nevertheless remained incomplete and varied across farms and recommendation types. The randomized assignment provides a basis for evaluating reinforcement, although missing endline outcomes limit the complete-case comparison. The separate analysis of achieved compliance identified a strong association with endline lameness prevalence, but did not establish a causal effect of compliance.

The reinforcement visit combined review of completed work, reminders and discussion of implementation difficulties. Any of these activities may have contributed to the observed difference in compliance, but the study did not measure their separate effects. Motivation, accountability and sustained attention remain possible explanations rather than demonstrated mechanisms. Research on agricultural extension and veterinary feedback supports evaluating these processes in future studies (Blekking et al., 2026; Kondylis et al., 2017; Ritter et al., 2017, 2019; Sumner et al., 2020).

From a practical standpoint, reinforcement plausibly functioned as a sustaining mechanism in this RCT, supporting continued attention to recommended actions between visits, rather than simply increasing knowledge. Indeed, this perspective aligns with qualitative evidence from large scale dairy systems reporting that even when producers are generally willing to adopt recommendations, implementation would be commonly limited by staffing, motivation of workers and constraints posed by existing infrastructure (Douay–Ryckelynck et al., 2025). While this large-scale context may differ from the smallholder dairy observed in this study, the shared theme is that the binding constraint is often sustained execution rather than initial awareness.

### Item-level compliance profile and feasibility constraints

Implementation varied across recommendation types, indicating that uptake was uneven across the recommended package. Across intervention farms, the proportion recommended varied markedly by item (e.g., body length, neck rail, and brisket board were recommended to most farms; others to relatively few SDFs), and partial/full implementation was similarly uneven. The within-farm paired midline-to-endline changes among farms observed at both time points further suggested that compliance is not fixed: many items showed net movement towards at least partial implementation.

Implementation varied across recommendation types, and the paired summaries showed changes between midline and endline among reinforcement recipients. Differences in cost, labour, feasibility or perceived benefit may have contributed to these patterns, but the item-level analyses did not identify the reasons for implementation. Earlier Kenyan studies also reported differences by recommendation type, although the direction of the association with major versus minor effort was not consistent across studies. Kathambi et al. (2019) reported higher implementation odds for major recommendations, whereas Kariuki et al. (2023) reported higher odds for minor recommendations.

### Reinforcement assignment and animal outcomes

In the analysis by randomized reinforcement assignment, reinforcement was associated with higher milk yield at endline (+1.33 kg/day; 95% CI 0.32 to 2.35; p = 0.011) and with a lower prevalence of lameness (−19.75 percentage points; 95% CI −33.55 to −5.96; p = 0.005). These results are directionally consistent with the cow comfort literature that links improved stall conditions to reduced lameness and improved performance (von Keyserlingk et al., 2012). Notably, lower prevalences of some stall deficits were also observed among reinforcement-assigned farms. These concurrent findings do not establish that stall changes mediated the lameness or milk-yield differences, because mediation was not analysed.

In contrast, several health and hygiene-related welfare indicators showed limited to no evidence of change under reinforcement, including subclinical mastitis (negative percentage point estimates but imprecise) and leg, udder and stall hygiene measures (near-zero contrasts with wide confidence intervals). This pattern is plausible in a setting where the farm-specific recommendation package and compliance score emphasize cow comfort recommendations: improvements in stall design may affect lameness and potentially yield over a relatively short horizon, whereas detectable shifts in subclinical mastitis prevalence may require stronger mastitis-specific implementation recommendations (e.g., milking hygiene, teat disinfection, dry-cow strategies) and/or longer follow-up. Consistent with this viewpoint, a related trial in Kenya that explicitly bundled cow comfort and mastitis control recommendations discussed mastitis outcomes in relation to compliance with mastitis-specific measures (Ng’ang’a et al., 2025).

### Linking compliance to outcomes

Because compliance is a post-randomization behaviour, estimating its association with welfare/productivity required consideration of confounding by farm capacity, management and baseline comfort status. In the weighted analysis, high compliance (≥40%) was associated with a lower endline lameness prevalence (−49.39 percentage points; 95% CI −60.64 to −38.15; p < 0.001), while the corresponding milk yield contrast, though positive, was not statistically significant (p = 0.121).

The magnitude of the lameness association requires caution. Compliance and lameness were assessed at endline, and individual cows could not be consistently linked across visits. The analysis therefore cannot establish that implementation preceded improvement in locomotion in the same animals. Within-farm prevalences were also based on small numbers of assessed cows, with a baseline median of two. Changes in the animals assessed could consequently produce large changes in these proportions. Differences in farm management, resources, hoof care, feeding or other unmeasured determinants of both compliance and lameness may have contributed to the association. Improved housing conditions provide a plausible explanation for better locomotion, but the present study did not identify the causes of lameness. Agreement between the linear and fractional-logit estimates shows consistency across two outcome specifications; both nevertheless use the same observations, exposure definition and weighting approach.

The reported weights had a limited upper tail, which supports the numerical stability of the fitted weighted analyses. As observed by Cole and Hernan (2008), this limited upper tail of the weights suggests that individual farms were not assigned disproportionately large weights, though it does not eliminate possible residual confounding by unmeasured determinants of both compliance and outcomes (e.g., unmeasured managerial skills, household liquidity shocks, etc.). The comparison in Table 9 changes both censoring adjustment and truncation, so it does not isolate the contribution of censoring adjustment.

### Internal validity considerations

Endline completion was 91.5% among farms assigned to reinforcement and 83.0% among farms assigned to no reinforcement. This difference can affect analyses restricted to observed outcomes even though the comparison of completion proportions did not reach the conventional 0.05 threshold. Censoring weights address selection only to the extent that the observation model includes the relevant predictors and is appropriately specified. Selection related to unmeasured determinants of follow-up and outcomes remains possible.

### Limitations

Several limitations should be considered when interpreting these findings. Firstly, the study relied on repeated cow assessments, but cow identifiers were not consistently stable across survey rounds, which prevented robust linkage of the same animal across time. As a result, the primary welfare and stall endpoints measured at the cow level were summarized as farm-level prevalences rather than analyzed as cow-level longitudinal outcomes. Although this approach was sensible since the unit of randomization was the farm, it reduced the ability to estimate within-cow changes, and the ability to model heterogeneity of intervention effects across cows within farms. Future longitudinal research involving smallholder farmers in Kenya should incorporate unique, persistent cow identifiers (e.g., ear tags) to enable mixed-effects models that explore repeated cow measures while still accounting for farm-level clustering.

Secondly, compliance assessment was subject to judgement, particularly when distinguishing partial from full implementation. The same observers were used throughout the study, but formal observer agreement was not quantified. Measurement error in the composite compliance score could affect the estimated associations, and its direction cannot be assumed to be toward the null. The absence of formal score validation also limits interpretation of differences between farms. In other words, the weighted compliance score has not been formally validated, and the ≥40% classification discards variation within the two compliance categories. Farms with similar scores may also have implemented different recommendations. These features limit interpretation of the score as a uniform level of exposure to improved cow comfort.

Finally, generalizability may be constrained by the specific smallholder context (≤5 cows; zero-/semi-zero-grazing systems in Uasin Gishu County) and by the nature of the recommendation package. Some structural recommendations are capital-intensive and may be harder to implement in other contexts with different resource constraints, materials access, or housing typologies. Future work should evaluate reinforcement strategies across additional production systems and test complementary enabling interventions (e.g., low-cost stall design kits, microfinance linkages) where implementation barriers have been established.

### Implications for policy and future research

First, reinforcement appears to be a good implementation strategy that can be leveraged for improving uptake of welfare-relevant husbandry changes in smallholder settings, especially where recommendations include feasible (low-cost), or can be paired with enabling support. Existing evidence reports that strengthening follow-up and engagement can improve adoption behaviours (Blekking et al., 2026; Kondylis et al., 2017). Second, the item-level profile indicates that structural modifications remain difficult even under reinforcement, thus future programs may need to pair behavioural reinforcement with enabling inputs (e.g., low-cost design templates, linkages to service providers, subsidies or microfinance mechanisms) if the objective is to shift the most capital-intensive adjustments.

Future research could improve the methodology of the study by: (i) extending the follow-up period to better capture gradual adopters’ compliance or slower-moving health outcomes, such as mastitis dynamics; (ii) testing alternative reinforcement approaches (e.g., SMS-only, versus in-person versus hybrid) and their cost-effectiveness; and (iii) examine compliance as a continuous exposure and assess sensitivity to alternative classifications.

## Conclusions

Among intervention farms with observed and defined endline compliance scores, assignment to midterm reinforcement was associated with higher implementation of farm-specific cow-comfort recommendations. Analyses by reinforcement assignment also showed higher endline milk yield and lower lameness prevalence, although incomplete follow-up limits these comparisons. In the separate weighted analysis, higher achieved compliance was associated with lower endline lameness prevalence; the milk-yield association was not statistically significant. Because compliance was not randomized and was assessed alongside the endline outcomes, these associations do not establish that higher compliance caused the observed differences. These findings support reinforcement as a useful strategy to strengthen adoption of welfare-improving recommendations in the study setting.

## Ethical approval

This study was approved by the Conjoint Faculties Research Ethics Board (CFREB) - REB24–0284 - and Animal Care Committee (AC24 – 0043) of the University of Calgary. It was also reviewed and approved by the Research Ethics Board and Animal Care Committee of the University of Prince Edward Island (REB Ref# 6012,044). Additionally, it was licensed in Kenya by the National Commission for Science, Technology and Innovation under license number NACOSTI/P/24/34,410. Prior to each farmer recruitment and interview, the study’s objectives, design and respondents’ rights were explained, a written consent to participate was obtained, and a signed copy of the consent form was retained by each party. On every follow-up visit, farmers were given the opportunity to confirm or withdraw their consent to participate in the study.

## Ethics declaration

Written informed consent to take part in the study and to publish the article has been obtained from all participants or their legal representatives. The privacy rights of participants have been observed.

This study was performed in compliance with relevant laws, regulatory frameworks and guidelines where the research took place. This study was approved by the Conjoint Faculties Research Ethics Board and Animal Care Committee of the University of Calgary Research Ethics Board and Animal Care Committee of the University of Prince Edward Island. (Approval No REB24–0284; REB Ref# 6012,044)

This study was conducted in accordance with the ARRIVE (Animal Research: Reporting of In Vivo Experiments) guidelines. This study was approved by the Conjoint Faculties Research Ethics Board and Animal Care Committee of the University of Calgary. (Approval No AC24 – 0043)

## Funding sources

This research was funded by the Sir James Dunn Animal Welfare Centre (University of Prince Edward Island). The work also received financial and material support from ILRI through the One CGIAR Research Program on Sustainable Animal Productivity for Livelihoods, Nutrition and Gender Inclusion (SAPLING initiative) funded by the CGIAR Trust Fund. The results and conclusions contained in this publication are solely those of the authors and do not necessarily reflect the policies or views of the funders or the authors’ affiliate institutions.

## CRediT authorship contribution statement

**Emmanuel Muunda:** Writing – original draft, Validation, Software, Resources, Project administration, Methodology, Investigation, Formal analysis, Data curation, Conceptualization. **Elizaphan James O Rao:** Writing – review & editing, Supervision, Project administration, Investigation, Funding acquisition, Formal analysis, Conceptualization. **David C Hall:** Writing – review & editing, Supervision, Software, Resources, Project administration, Funding acquisition, Data curation, Conceptualization. **Caroline Ritter:** Writing – review & editing, Methodology, Investigation, Data curation, Conceptualization. **Maria Camila Ceballos:** Writing – review & editing, Validation, Investigation, Conceptualization. **John VanLeeuwen:** Writing – review & editing, Supervision, Project administration, Methodology, Investigation, Funding acquisition, Formal analysis, Data curation, Conceptualization.

## Declaration of competing interest

The authors declare that they have no known competing financial interests or personal relationships that could have appeared to influence the work reported in this paper.

## Footnotes

1 All 106 control farms had a within-farm proportion of 1.00, and the group-specific mean-estimation command returned missing confidence limits.
